# Artemis inhibition as a therapeutic strategy for acute lymphoblastic leukemia

**DOI:** 10.3389/fcell.2023.1134121

**Published:** 2023-03-31

**Authors:** Heather A. Ogana, Samantha Hurwitz, Chih-Lin Hsieh, Huimin Geng, Markus Müschen, Deepa Bhojwani, Mark A. Wolf, James Larocque, Michael R. Lieber, Yong Mi Kim

**Affiliations:** ^1^ Department of Pediatrics, Children’s Hospital Los Angeles, Division of Hematology and Oncology, Keck School of Medicine, University of Southern California, Los Angeles, CA, United States; ^2^ Department of Urology, USC Norris Comprehensive Cancer Center, Keck School of Medicine, University of Southern California, Los Angeles, CA, United States; ^3^ Department of Laboratory Medicine, UCSF, San Francisco, CA, United States; ^4^ Department of Immunobiology, Center of Molecular and Cellular Oncology, Yale University, New Haven, CT, United States; ^5^ Curia Global Inc, Albany, NY, United States; ^6^ Departments of Pathology, The Molecular and Computational Biology Section of the Department of Biological Sciences, USC Norris Comprehensive Cancer Center, Biochemistry and Molecular Biology, Molecular Microbiology and Immunology, Keck School of Medicine, University of Southern California, Los Angeles, CA, United States

**Keywords:** acute lymphoblastic leukemia, ARTEMIS, pharmacological inhibition, proliferation, V(D)J recombination, DNA hairpin, double-strand break, SNM1 nucleases

## Abstract

As effective therapies for relapse and refractory B-cell acute lymphoblastic leukemia (B-ALL) remain problematic, novel therapeutic strategies are needed. Artemis is a key endonuclease in V(D)J recombination and nonhomologous end joining (NHEJ) of DNA double-strand break (DSB) repair. Inhibition of Artemis would cause chromosome breaks during maturation of RAG-expressing T- and B-cells. Though this would block generation of new B- and T-cells temporarily, it could be oncologically beneficial for reducing the proliferation of B-ALL and T-ALL cells by causing chromosome breaks in these RAG-expressing tumor cells. Currently, pharmacological inhibition is not available for Artemis. According to gene expression analyses from 207 children with high-risk pre-B acute lymphoblastic leukemias high Artemis expression is correlated with poor outcome. Therefore, we evaluated four compounds (827171, 827032, 826941, and 825226), previously generated from a large Artemis targeted drug screen. A biochemical assay using a purified Artemis:DNA-PKcs complex shows that the Artemis inhibitors 827171, 827032, 826941, 825226 have nanomolar IC50 values for Artemis inhibition. We compared these 4 compounds to a DNA-PK inhibitor (AZD7648) in three patient-derived B-ALL cell lines (LAX56, BLQ5 and LAX7R) and in two mature B-cell lines (3301015 and 5680001) as controls. We found that pharmacological Artemis inhibition substantially decreases proliferation of B-ALL cell lines while normal mature B-cell lines are not markedly affected. Inhibition of DNA-PKcs (which regulates Artemis) using the DNA-PK inhibitor AZD7648 had minor effects on these same primary patient-derived ALL lines, indicating that inhibition of V(D)J hairpin opening requires direct inhibition of Artemis, rather than indirect suppression of the kinase that regulates Artemis. Our data provides a basis for further evaluation of pharmacological Artemis inhibition of proliferation of B- and T-ALL.

## Highlights

• Artemis gene expression is associated with poor survival in pediatric B-cell acute lymphoblastic leukemia (B-ALL).
• Pharmacological Artemis inhibition can decrease proliferation of patient-derived primary B-ALL cell lines.

## Introduction

V(D)J recombination is the gene rearrangement process by which the variable domain exons of the antigen receptors are assembled at the DNA level (Schatz and Swanson, 2011). The process is initiated by the RAG1/2 complex to generate DNA hairpins at the termini of the V, D, or J sub-exons. The DNA hairpins must be nicked before they can be ligated together to form the complete exon of the B and T cell antigen receptor. These DNA hairpins are unique in the genome and are absent in mature B and T cells and absent in all nonlymphoid cells.

Artemis, a DNA nuclease in the metallo-β-lactamase family, is an enzyme that is specifically responsible for opening these DNA hairpins (Moshous et al., 2001; Lieber, 2010). Therefore, inhibition of Artemis to block its hairpin opening action would result in double-strand DNA (chromosome) breaks in pro-B/pre-B and pro-T/pre-T cells (Watanabe et al., 2022). Artemis is an ideal therapeutic target because it is a structure-specific nuclease which, upon inhibition, could result in the persistence of chromosome breaks. This is because the V(D)J recombination activating gene (RAG) enzyme complex generates DNA hairpins at T-cell receptor and immunoglobulin gene loci, which must be processed by Artemis to allow ligation and maintain chromosome integrity (Kurosawa and Adachi, 2010; de Villartay, 2015; Felgentreff et al., 2015; Betermier et al., 2020; Shibata and Jeggo, 2020; Fournier et al., 2022).

One strategy that we have been pursuing for targeting ALL cells takes advantage of the fact that over 90% of ALL patient cell lines express the RAG complex (Bories et al., 1991). Therefore, blocking Artemis systemically would cause chromosome breaks in ALL cells (Anne-Esguerra et al., 2022), resulting in inhibition of cell proliferation.

This rationale was supported here by examining the expression level data for Artemis in primary and relapse human ALL patients. Based on this rationale, a high throughput screen was done using a proprietary library to evaluate inhibition of the Artemis catalytic domain and its nuclease activity (to be reported elsewhere). Following hit confirmation and medicinal chemistry optimization to improve potency and selectivity, four compounds were assessed for inhibition of primary and relapse ALL cells.

## Methods

### Patient samples and cells

Bone marrow (BM) and peripheral blood (PB) samples from ALL patients were acquired in compliance with the Institutional Review Board regulations of each institution. Informed consent for cell banking was obtained from all human subjects. Leukemia cells were processed and cultured as previously described (Gang et al., 2020). Human studies were conducted in accordance with the Declaration of Helsinki.

Normal mature human B cell lines, immortalized lymphoblastoid cell lines 3301015 and 5680001, were generated using EBV-transformation of peripheral blood from patients with no hematopoietic abnormalities.

## Correlation of Artemis gene expression on leukemic blasts with clinical outcomes of pre-B ALL patients

**FIGURE 1:**
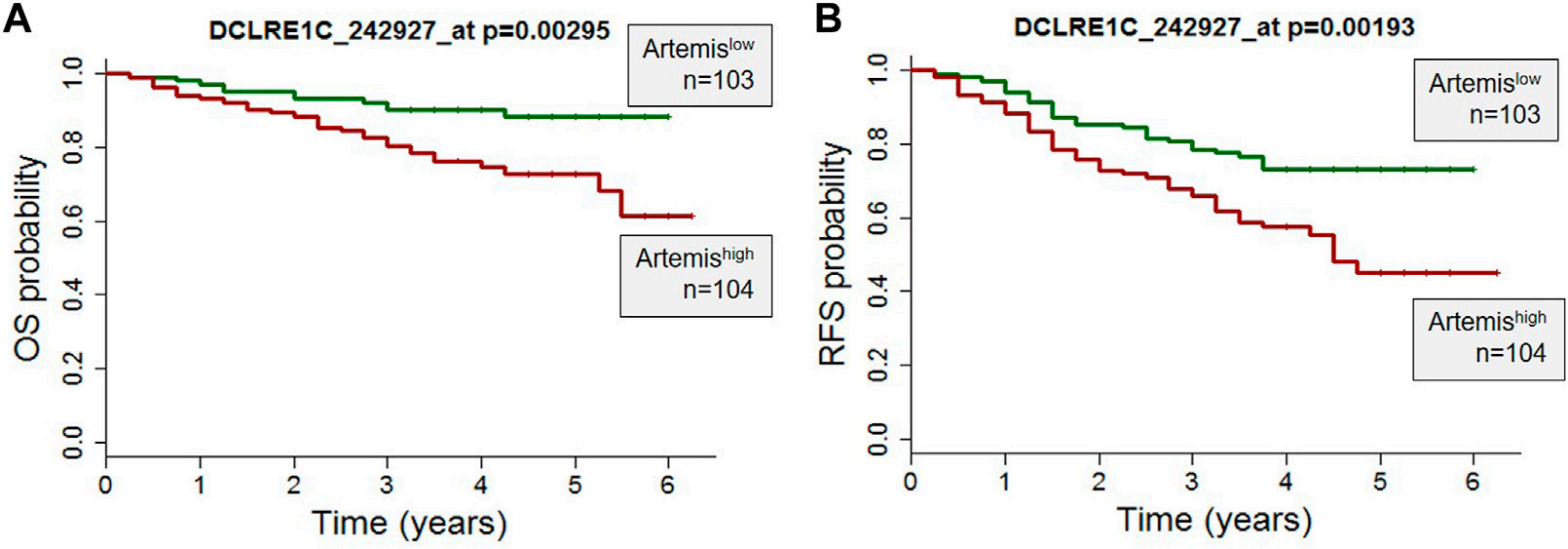
High Artemis expression is associated with poor outcome in primary B-ALL. **(A)** A probe set for Artemis in 207 pediatric ALL cases from COG study P9906 were analyzed. RNA was purified from 207 pretreatment diagnostic samples with more than 80% blasts (131 BM, 76 PB). **(A)** Overall Survival Probability (OS) and **(B)** Relapse Free Survival (RFS) of Artemis low (*n* = 103) and Artemis high (*n* = 104) are shown. *p*-values were obtained from the Wilcoxon test for each probe set (*p = 0.00295 and *p=0.00193 for probe 242927_at).

Clinical and gene expression microarray data from 207 high-risk B-precursor ALL patients from the COG Clinical Trial P9906 were obtained from the GEO database (GSE11877) (Kang et al., 2010). The patients were treated uniformly with a modified augmented Berlin-Frankfurt-Münster Study Group (BFM) regimen, and individuals with very high-risk features (*BCR-ABL1* or hypodiploidy) were excluded from the study (Figure 1). Cryopreserved residual pretreatment leukemia specimens were available for a representative cohort of 207 patients, including 131 BM and seventy-six peripheral blood (PB) samples. Artemis expression (probeset DCLRE1C_242927) was determined by Affymetrix GeneChip analyses. The correlation of Artemis with overall survival analysis (OS) and relapse free survival (RFS) analysis was performed using the Wilcoxon test in the R package (R Development Core Team http://www.R-project.org/).

### *In vitro* drug assay

Lentivirally green fluorescence protein (GFP) labeled patient-derived B-ALL cells (LAX56, BLQ5, LAX7R) or mature B cell lines 3301015 and 5680001 were plated on irradiated murine stromal OP9 cells and treated with four compounds optimized for Artemis inhibition (827171, 827032, 826941, 825226) (provided by Curia Global Inc., as part of the NCI NExT Program). These compounds ranged in K_D_ binding affinity to Artemis from 20 to 150 nM. The commercially available DNA-PK inhibitor (AZD7648) (obtained from a commercial source) was evaluated in parallel as a positive control. Proliferation was determined by measuring the green fluorescence intensity per image (9 images were captured per well) by Incucyte every 8 h for 3 days. Raw values were normalized to the zero-time DMSO control. On day 3, viability of the treated cells was determined by annexin V/DAPI staining *via* flow cytometry.

### Biochemical Artemis catalytic activity assay

Artemis activity assay was developed using full length Artemis with DNA-PKcs to generate a fluorescent signal from the cleavage of the 5′ 6-FAM modified end of hairpin DNA with a black-hole quencher (3-BHQ) on the 3’ end. The assay buffer used was 25 mM MOPS, pH 7.5, 10 mM MgCl2, 10 mM KCl, 10 μM ATP, 1 mM DTT, 0.05% (w/v) CHAPS with 5 nM FL-Artemis, 5 nM DNA-PKcs, and 200 nM DNA FAM hairpin substrate. The fluorescence signal was monitored kinetically on a Perkin Elmer Envision microplate reader with FITC filters (*λ*_ex_ = 485 nm, *λ*_em_ = 520 nm) with 1-min reads for 40 min. Compounds were tested in 10-point concentration (10 different concentrations with 3-fold dilutions) responses using the slopes of the enzyme readouts to determine percent inhibition which was fit to a 4-parameter logistic curve to calculate the IC_50_ of each compound. We have previously published the purity of the protein preparations (Ma et al., 2002; 2004; Lu et al., 2008), and these will be published once again as part of the high throughput study of the large chemical libraries used.

## Results

### Artemis expression in patient B-ALL cells inversely correlate with clinical outcome

To determine the role of Artemis in ALL, expression of Artemis mRNA (DCLRE1C) in 207 ALL patients uniformly treated with the COG P9906 clinical trial (Kang et al., 2010) was correlated with outcome. Artemis expression was determined by Affymetrix GeneChip analyses on ALL tumor cells at the time of diagnosis (prior to chemotherapy). The overall survival (OS) (Figure 1A) and relapse free survival (Figure 1B) were analyzed by Artemis expression and could be separated into Artemis^high^ (DCLRE1C expression ≥mean; *n* = 104) and Artemis^low^ expressing cases (DLCRE1C expression <mean, *n* = 103). Artemis^high^ leukemias were associated with inferior outcome with lower overall survival and relapse-free survival. This supported the rationale for targeting Artemis.

### Specificity of the Artemis inhibitors

**FIGURE 2:**
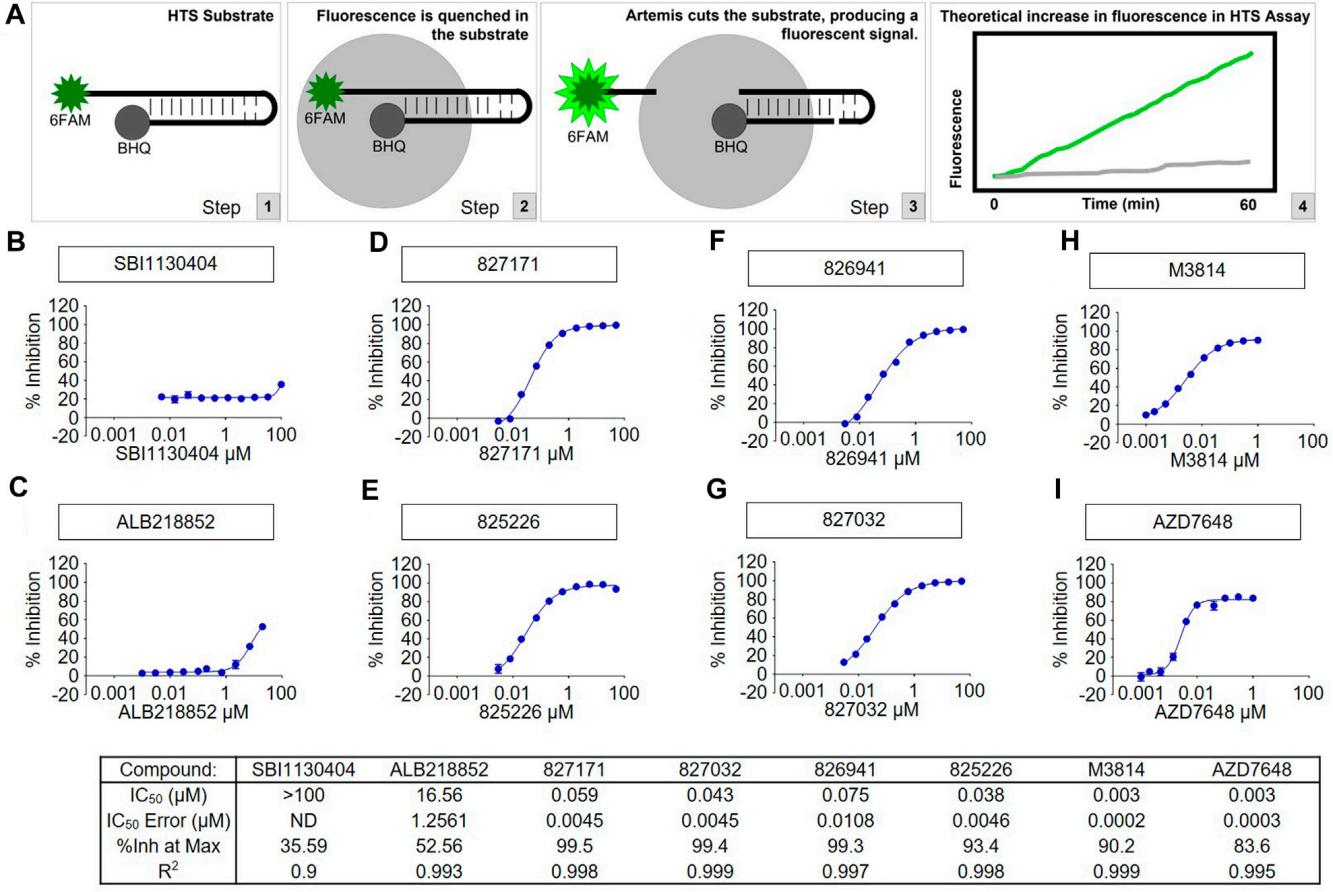
The tested compounds are potent inhibitors of the catalytic activity of purified full-length human Artemis with DNA-PKcs. **(A)** Schematic of High-Throughput Screening (HTS) Assay. Steps 1, 2, and 3 show the fluorescence assay for cutting of the DNA hairpin substrate by Artems:DNA-Kcs. Artemis activity assay was optimized for development of a fluorescent signal from the cleavage of the 6-FAM modified end of hairpin DNA (to release it from proximity to the BHQ quenching) and monitored kinetically on a Perkin Elmer Envision microplate reader with FITC filters (excitation wavelength = 485 nm, emission wavelength = 520 nm). Compounds were tested in concentration response and the slopes of the enzyme readouts were converted to percent inhibition fit to a 4-parameter logistic curve to calculate the IC_50_ of each compound. Two inactive compounds **(B)** SBI1130404 and **(C)** ALB218852, are shown for comparison. The assay was used to test compounds to aid in determining the structure activity relationship to develop through medicinal chemistry the Artemis inhibitors **(D)** 827171, **(E)** 827032, **(F)** 826941 and **(G)** 825226. The concentration responses curves and calculated results of the inhibitors are shown compared with known DNA-PK inhibitors **(H)** M3814 and **(I)** AZD7648.

A high throughput screen of Artemis active site inhibitors using a proprietary library to assess for inhibition of the Artemis catalytic domain and its nuclease activity identified four small molecule inhibitors (Figure 2A). The medicinal chemistry and high throughput screen that led to these will be described elsewhere. We determined the specificity of the four Artemis inhibiting compounds using a biochemical Artemis activity assay which generates a fluorescent signal from the cleavage of the 5′ 6-FAM modified end of hairpin DNA with a black-hole quencher (3-BHQ) on the 3’ end. For comparison, the AZD7648 and M3814 compounds were used to inhibit DNA-PKcs kinase activity. DNA-PKcs kinase activity is required for DNA-PKcs to autophosphorylate itself, and this autophosphorylation event thereby activates Artemis nuclease activity. Though Artemis activation as a nuclease predominantly requires DNA-PKcs kinase activity, direct inhibition of Artemis avoids the non-specific toxicity of generic kinase inhibitors that act at the ATP binding pocket of kinases. Two inactive compounds, SBI1130404 (Figure 2B) and ALB218852 (Figure 2C), are shown for comparison. Therefore, the compounds 827171, 827032, 826941, and 825226 (Figures 2D–G) act with nanomolar IC50 values to inhibit Artemis, and these are nearly as potent as the established commercial DNA-PKcs inhibitors, M3814 and AZD7648 (Figure 2H, I).

We have previously shown that full-length Artemis under Mg^2+^ conditions is entirely inactive without DNA-PKcs (Ma et al., 2002; 2004; Lu et al., 2008). This indicates that the Artemis:DNA-PKcs complex is formed; otherwise, no FAM hairpin substrate would be cut to generate the fluorescence signal.

### Evaluation of Artemis inhibitors on viability of B-ALL and mature B cell lines

**FIGURE 3:**
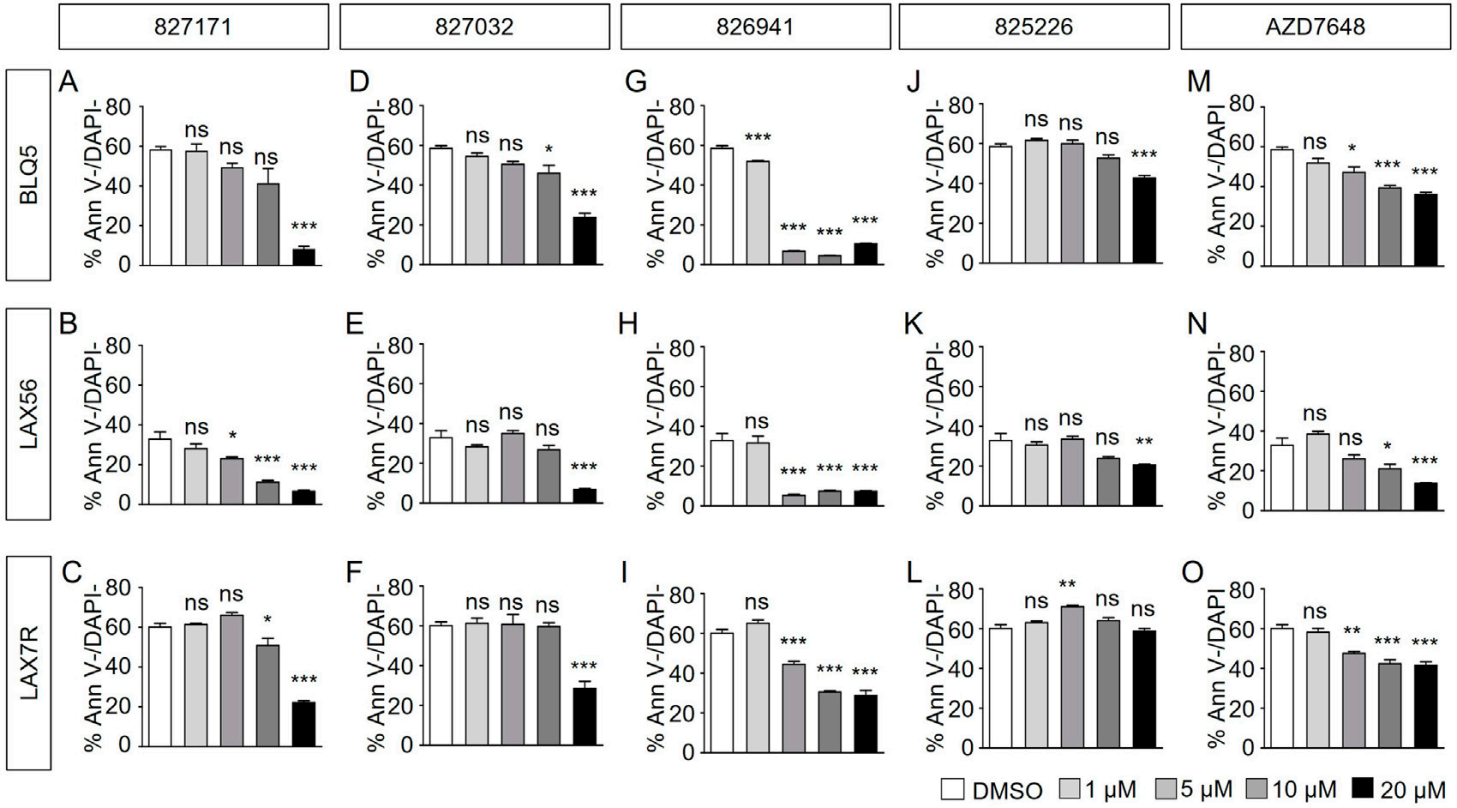
Artemis inhibitors and DNA-PK inhibitor AZD7648 effects on viability of B-ALL cells. B-ALL cells (BLQ5, LAX56 and LAX7R) were treated with Artemis inhibitor 827171 **(A–C)**, 827032 (D-F), 826941 **(G–I)**, 825226 **(J–L)** and DNA-PK inhibitor AZD7648 **(M–O)** with indicated concentrations. On day three, primary B-ALL cells were collected and used for flow cytometry analysis with PE Annexin V and DAPI to measure % viability (PE Annexin V-/DAPI-). The statistical analysis of viability differences is compared to DMSO control: ns: non-significant; *p < 0.05; **p < 0.001; ***p < 0.0001, one-way ANOVA.

Here, we determined the effect of four Artemis inhibitors 827171 (Figures 3A–C), 827032 (Figures 3D–F), 826941 (Figures 3G–I), 825226 (Figures 3J–L) and the DNA-PK inhibitor AZD7648 (Figures 3M–O) on the viability of patient-derived B-ALL cells (BLQ5, LAX56 and LAX7R) (Table 1) after an incubation time of 3 days. Three of four Artemis inhibitors, 827171 (Figures 3A–C), 827032 (Figures 3D–F), 826941 (Figures 3G–I), showed a dose-dependent mild to moderate decrease in viability in all three tested B-ALL cells compared to DMSO controls. Of these three compounds, compound 827171 showed the smallest effect on viability. The Artemis inhibitor 827032 (Figures 3D–F) only showed a decrease in viability at 20 μM in all three B-ALL cases. The compound, 825226 (Figures 3J–L), only showed a dose-dependent decrease in viability in BLQ5 and LAX56 cells, but not in LAX7R cells, as 20 μM did not significantly affect viability. The compound 826941 affected the viability of all 3 cell lines at doses 5–20 μM. All cell lines showed a decrease in viability after exposure to the reference control DNA-PK inhibitor AZD7648 (Figures 3M–O).

**TABLE 1:** Patient-derived B-ALL information.

B-ALL cells	Diagnosis	Cytogenetics
BLQ5	Relapse	BCR-ABL1
LAX56	Relapse	t(Y;7)(p1.3;p13)
LAX7R	Relapse	KRASG12V

**FIGURE 4:**
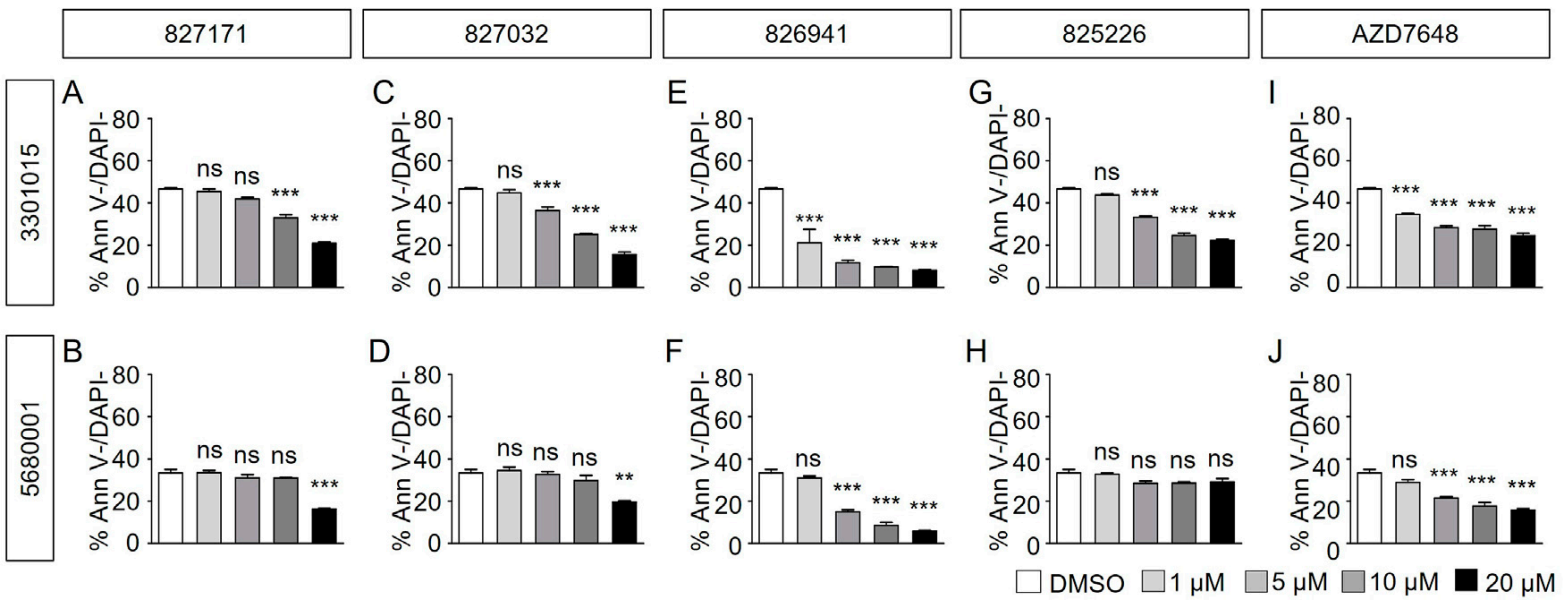
Artemis inhibitors and DNA-PK inhibitors effect on viability of normal mature B cell lines. Normal mature B cell lines 3301015 and 5680001 were treated for 3 days with 827171 **(A, B)**, 827032 **(C, D)**, 826941 **(E, F)**, 825226 **(G, H)** and DNA-PK inhibitor AZD7648 **(I, J)** with indicated concentrations. On day three, mature B cells were collected and used for flow cytometry analysis with PE Annexin V and DAPI to measure % viability (PE Annexin V-/DAPI-). The % viability (Ann V-/DAPI-) is depicted. The statistical analysis of viability differences is compared to DMSO control: ns: non-significant; *p < 0.05; **p < 0.001; ***p < 0.0001, one-way ANOVA.

Normal B cell lines 3301015 and 5680001 were treated for 3 days with Artemis inhibitors 827171 (Figures 4A, B), 827032 (Figures 4C, D), 826941 (Figures 4E, F), 825226 (Figure 4G, H). It is important to note that the viability of the normal mature B-cell lines is low in general (DMSO control). Artemis inhibitors 827171 (Figures 4A, B), 827032 (Figures 4C, D), 825226 (Figure 4G, H) do not affect viability of B cell lines 3301015 and 5680001 at 1 μM. However, 826941 (Figures 4E, F) and AZD7648 (Figures 4I, J) decrease viability of one or both B-cell lines at 1–20 μM. Similarly, AZD7648 (Figures 4I, J) decreases the viability of one or both B-cell lines at 1–20 μM. The statistical analysis of all viability differences is compared to DMSO controls.

Overall, the 827171 compound among the four has the smallest effects on B-ALL or mature B cell viability. Cells affected by 827171 may not decrease in viability but they may be arrested in S phase and therefore be slowed in the proliferation assay, as we investigated below (Wang et al., 2009; Yan et al., 2011).

### Artemis inhibitor 827171 substantially reduces proliferation of B-ALL compared to normal B-cell lines

**FIGURE 5:**
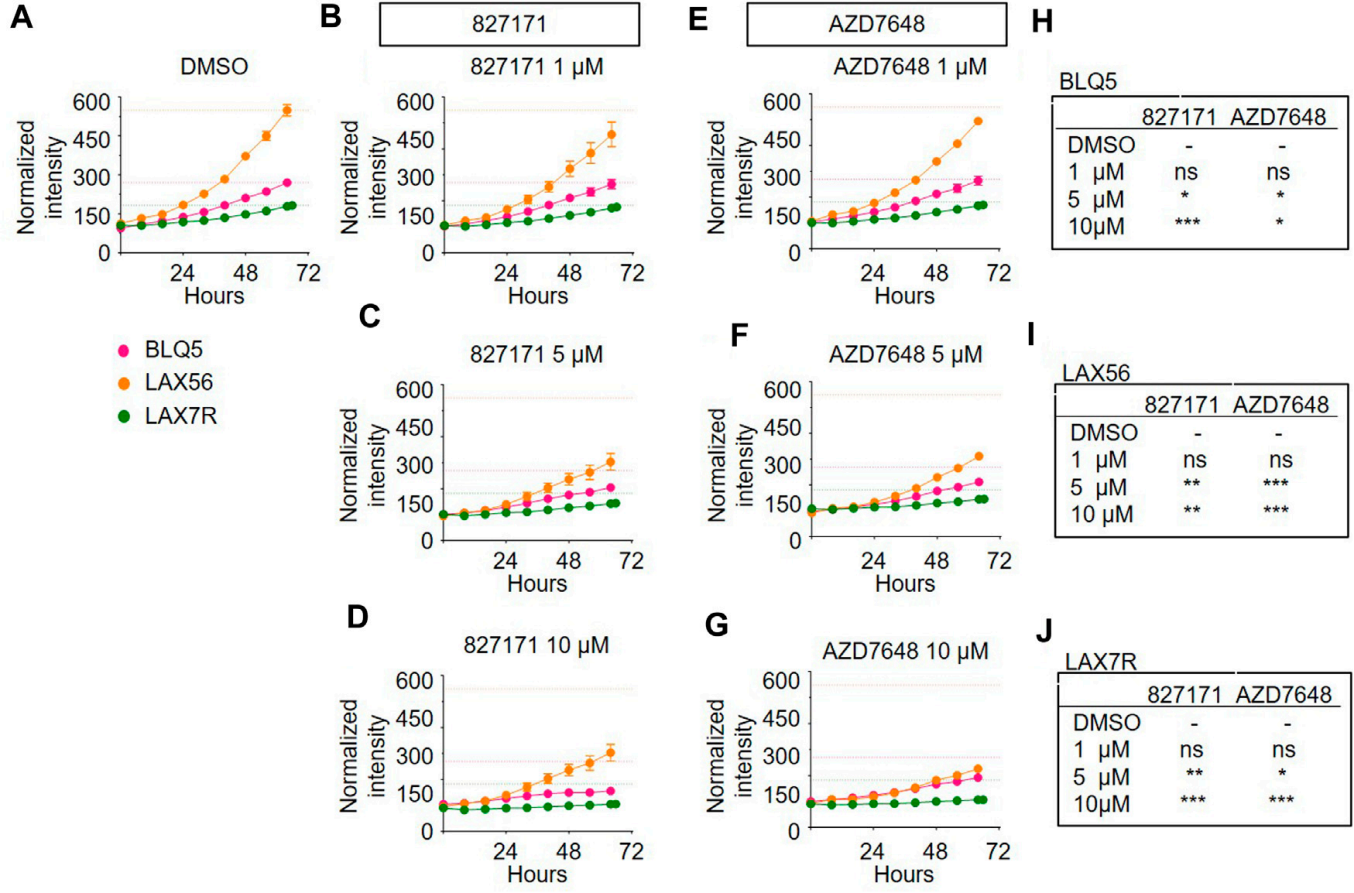
The Artemis inhibitor 827171 and DNA-PK inhibitor AZD7648 substantially inhibit proliferation of B-ALL. Primary B-ALL cells (BLQ5, LAX56, and LAX7R) were transduced with GFP, and 100,000 cells were plated in 24-well plates seeded with irradiated OP9 cells. Cells were treated with DMSO control **(A)**, Artemis inhibitor 827171 **(B–D)** or DNA-PK inhibitor AZD7648 **(E–G)** with the indicated doses. Green fluorescence intensity was measured by Incucyte every 8 h. Raw values were normalized to the 0 h DMSO control. The dashed lines indicate the mean of the final time point measurements of the DMSO control for each primary B-ALL cell. The statistical analysis of proliferation differences is shown for the final time point measurements for BLQ5 **(H)**, LAX56 **(I)**, LAX7R **(J)**, after treatment with 827171 or DNA-PK inhibitor AZD7648 at indicated doses compared to DMSO control. ns: non-significant. *p < 0.05; **p < 0.001; ***p < 0.0001, one-way ANOVA.

**FIGURE 6:**
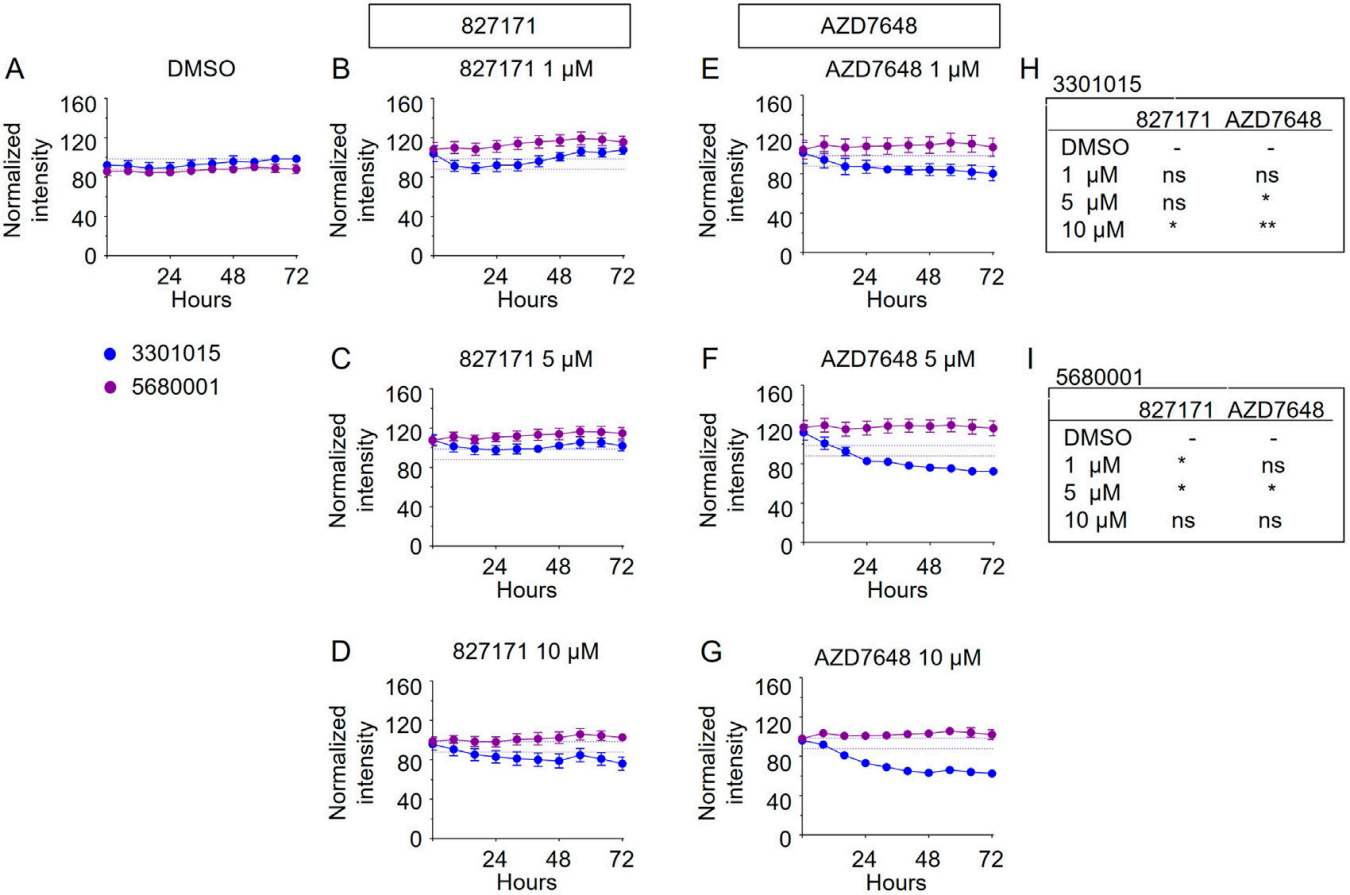
The Artemis inhibitor 827171 and DNA-PK inhibitor AZD7648 have minimal effect on mature B cells. Mature B cell lines (3301015 and 5680001) were transduced with GFP, and 100,000 cells were plated in 24-well plates seeded with irradiated OP9 cells. Cells were treated with DMSO control **(A)** or Artemis inhibitor 827171 **(B–D)** or DNA-PK inhibitor AZD7648 **(E–G)** with the indicated doses. Green fluorescence intensity was measured by Incucyte every 8 h. Raw values were normalized to the 0 h DMSO control. The dashed lines indicate the mean of the final time point measurements of the DMSO control for each mature B cell line. The statistical analysis of proliferation differences is shown for the final time point measurements for 3301015 **(H)** or 5680001 **(I)** after treatment with 827171 or DNA-PK inhibitor AZD7648 at indicated doses compared to DMSO control. ns: non-significant. *p < 0.05; **p < 0.001; ***p < 0.0001, for statistically significant changes in proliferation of a treatment group compared to DMSO by one-way ANOVA.

Based on these viability studies, we next tested the compounds for their effect on proliferation. Preliminary testing indicates that the 827171 compound showed the largest effect on proliferation, and it was chosen for more detailed study. The 827171 compound and AZD7648 were evaluated in parallel. Ideally, normal B-cell lines would continue to proliferate upon Artemis inhibition and only B-ALL cell lines would be inhibited due to chromosome breaks. Primary patient-derived B-ALL cells (BLQ5, LAX56, and LAX7R) were transduced with GFP and 100,000 cells were plated in 24-well plates seeded with irradiated OP9 cells. Increasing green fluorescence intensity is indicative of proliferation as measured by Incucyte every 8 h. Although the GFP expression varied between cell lines (Supplementary Figure S1), the normalized data show the relative changes in proliferation compared to the DMSO control, which showed continuous proliferation of B-ALL (Figure 5A). The mature B cell line proliferation is slow enough that it appears relatively flat in the proliferation assays, but the cells do increase in number (Supplementary Figure S2). Compared to B-ALL cells which grow as single cells (Supplementary Figure S3A–F), the mature B-cell lines grow in clusters and the measurement of total intensity per image is a potential caveat and may not capture this growth (Supplementary Figure S3G–J). Artemis inhibitor 827171 (Figures 5A–D) showed inhibition of B-ALL cell lines at 5 μM and 10 μM and no decrease in proliferation of normal B-cells (Figures 6A–D). The proliferation of LAX56 was also decreased, which was not observed after treatment with the other Artemis inhibitors described earlier. The DNA-PK inhibitor AZD7648 (Figures 5E–G) inhibited the proliferation of B-ALL cell lines at 5μM and 10 µM. AZD7648 also suppressed proliferation of the normal mature B cell 3301015 at 5μM and 10 µM. There were no significant decreases in proliferation for 5680001 in either 827171 or AZD7648, but rather the compounds at 1 or 5 μM may have caused a minor increase in proliferation. Statistical analysis is compared to DMSO control and is summarized in Figures 5H–J. Thus, the 827171 compound shows substantial inhibition of B-ALL cell proliferation and minimal effects on normal mature B-cells.

## Discussion

Our studies indicate that the 827171 compound substantially inhibits the proliferation of primary patient B-ALL lines, and it is comparable to the AZD7648 compound (Figure 5). The advantage of direct inhibition of the structure-specific Artemis endonuclease is that it avoids the typical complications of kinase inhibitors that block many ATP binding pockets of the >3,000 kinases in the cell.

The effects of 827171 on viability, in contrast to proliferation, are among the mildest of the compounds evaluated. This likely reflects that DSBs in nondividing cells may not cause death immediately or at all. In contrast, an unrepaired DSB in proliferating cells represents more of a challenge. This increased effect of DSBs on proliferating cells may be because of a much higher probability of losing large chromosome fragments. Such a loss is because only the portion of the chromosome with the centromere is retained at the metaphase plate during cell division, and the chromosomal fragment lacking a centromere portion is easily lost. In addition, inhibition of Artemis may interfere with the ability of cells to exit from S phase (Wang et al., 2009; Yan et al., 2011).

The large majority of acute lymphoblastic leukemias (ALL) produce the RAG enzyme complex encoded by the RAG genes (Bories et al., 1991). The RAG complex cuts DNA to generate a double-strand break (DSB) with a perfect DNA hairpin at the edge of each V, D, and J segment at heptamer/nonamer signal sequence sites. Off-target sites that have sequences sufficiently similar to heptamer/nonamer sites also are cut by the RAG complex at a lower, but still relevant frequency (Shimazaki et al., 2012; Papaemmanuil et al., 2014). The perfect DNA hairpins on each side of the double-strand break (DSB) must be nicked to open them before they are eligible for ligation and repair of the DSB. Artemis inhibitors would block hairpin opening, a key step in V(D)J recombination, resulting in chromosome breaks thereby killing the cells or slowing their growth. A side effect of this strategy would be the transient loss of the pro-B/pre-B and pro-T/pre-T cells in the bone marrow and thymus during the period of treatment, but the memory compartments of B and T cells would not be affected, and neither would the stem cells because none of these express the RAG complex.

Here, we evaluated four of the Artemis inhibitors generated from a high-throughput screen and medicinal chemistry effort. These were assessed in three primary human ALL samples, with human mature B cell lines used for comparison. For comparison with the Artemis inhibitors, we used a known DNA-PKcs inhibitor, AZD7648, because DNA-PKcs is a kinase which regulates Artemis activity, and this compound has been evaluated in clinical trials for utility in a variety of non-lymphoid human malignancies (Matsumoto, 2022).

We found that the 827171-compound reduced proliferation of the three B-ALL lines at 1, 5, and 10 µM without affecting the mature B-cell control lines. In contrast, the DNA-PKcs inhibitor, AZD7648, showed little inhibition of the B-ALL lines, and instead suppressed the growth of the mature B cell control lines. These findings are encouraging for future work to make derivatives of the 827171 compounds. Importantly, the DNA-PKcs inhibitor AZD7648 decreased the proliferation less than 827171 in these same lines. This suggests that inhibition of hairpin opening requires direct inhibition of Artemis, rather than indirect suppression of the kinase that regulates Artemis, namely, DNA-PKcs. This may be because Artemis can be activated by DNA ligase IV, even when DNA-PKcs is not present (Gerodimos et al., 2017). Therefore, inhibition of hairpin opening to cause chromosome breaks likely requires direct Artemis inhibition.

While the 827171 compound shows promise for inhibition of the active site of Artemis, the brief study here also raises the interest in parallel strategies for inhibiting Artemis. Future work may take advantage of the recently described cryo-EM structure for the basal state of the Artemis:DNA-PKcs complex to develop inhibitors that prevent Artemis from being activated (Watanabe and Lieber, 2022; Watanabe et al., 2022). Combinations of strategies to block hairpin opening and thereby create chromosome breaks in B-ALL cells is a promising approach.

## Data Availability

The original contributions presented in the study are included in the article/Supplementary Material, further inquiries can be directed to the corresponding authors.
