# Supplementary material for: Artemis inhibition as a therapeutic strategy for acute lymphoblastic leukemia
Source: Front Cell Dev Biol. 2023 Mar 31;11:1134121. doi: 10.3389/fcell.2023.1134121 (PMC10111164; doi:10.3389/fcell.2023.1134121)
Supplement: Supplementary file 1 [file DataSheet1.pdf]

Supplementary Figure 1

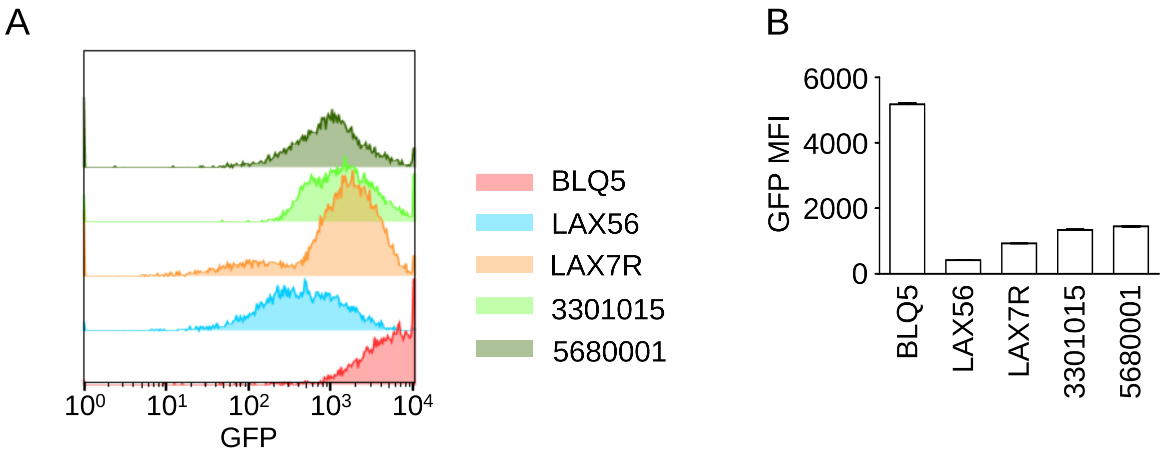

Supplementary Figure 2

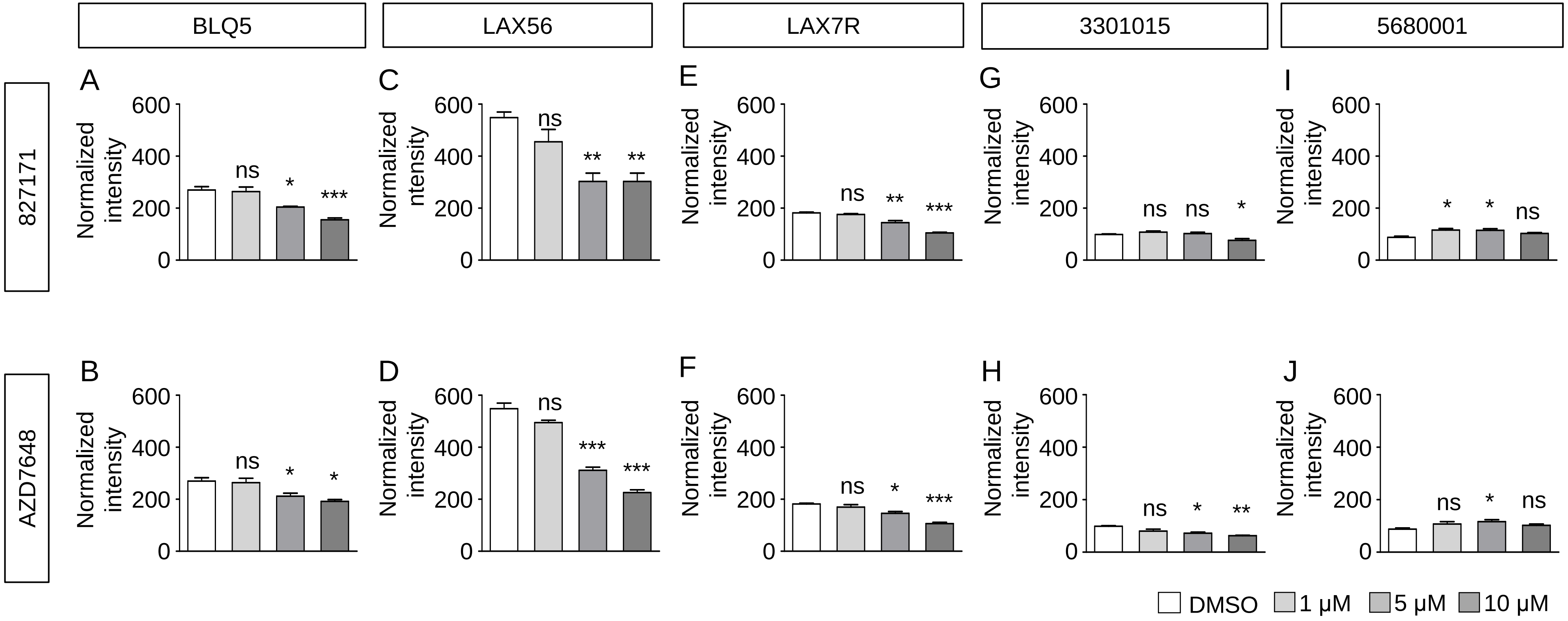

Supplementary Figure 3

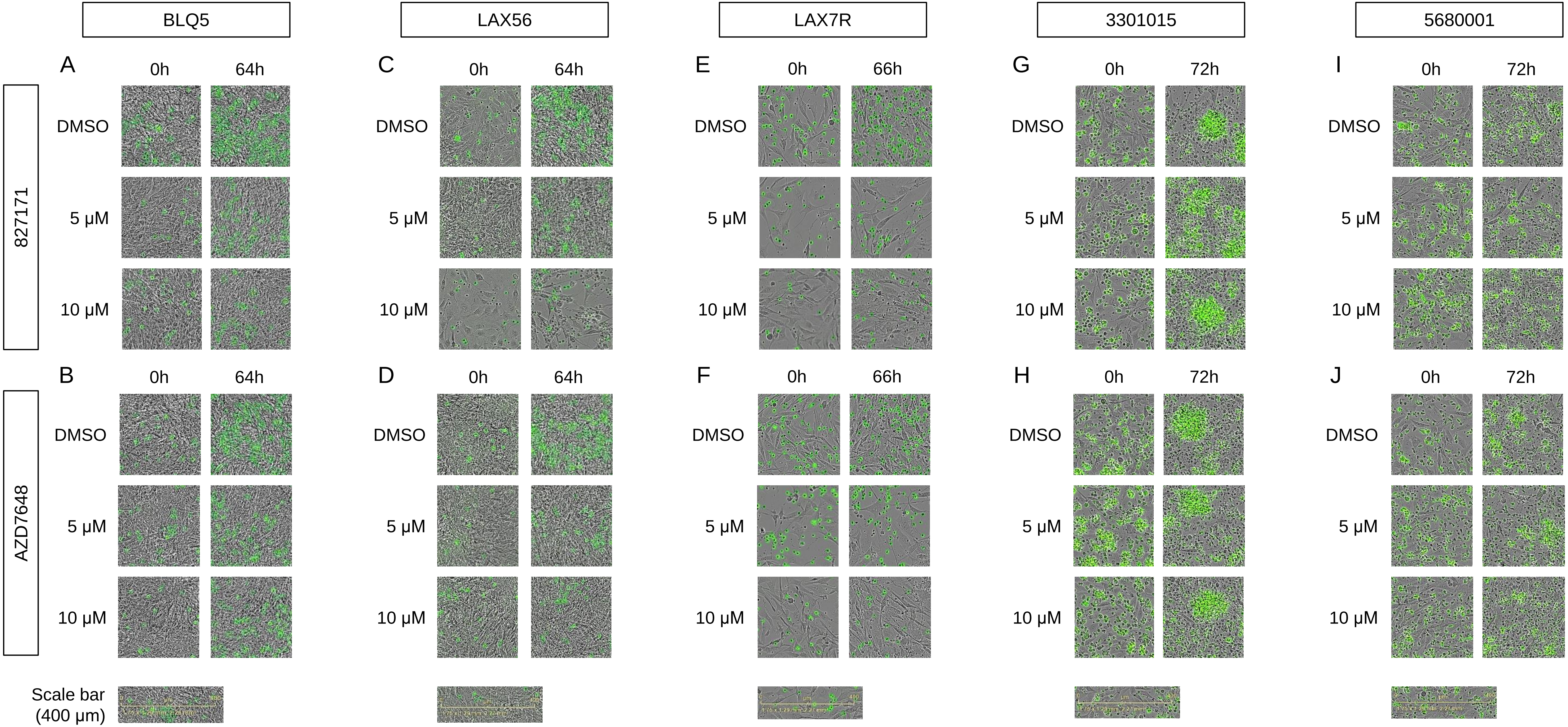

SUPPLEMENTARY MATERIAL

Supplementary Figure Legends

Supplementary Figure 1. The GFP expression per cell of the mature B cell lines is intermediate between the highest (BLQ5) and the lowest (LAX56) GFP-transduced cells. (A) GFP expression of representative DMSO-treated cells at the final time point for each cell line as detected by flow cytometry. B. Median fluorescence intensity (MFI) of GFP for DMSO-treated cells in triplicates for each cell line.

Supplementary Figure 2. The Artemis inhibitor 827171 and DNA-PK inhibitor AZD7648 significantly inhibit proliferation of B-ALL and have minimal effect on mature B cells. The statistical analysis of proliferation differences is shown for the final time point measurements from Figure 5 H-J and Figure 6 H-I. Normalized intensity is shown for BLQ5 (A, B), LAX56 (C, D), LAX7R (E, F), 3301015 (G, H) or 5680001 (I, J) after treatment with 827171 or DNA-PK inhibitor AZD7648, respectively, at indicated doses compared to DMSO control. *$p < 0.05$; **$p<0.001$; ***$p<0.0001$, for statistically significant changes in proliferation of a treatment group compared to DMSO by one-way ANOVA.

Supplementary Figure 3. Mature B cell lines grow in clusters while the B-ALL cells grow as single cells. Representative images captured by the Incucyte of cell lines BLQ5 (A, B), LAX56(C, D), LAX7R (E, F), 3301015 (G, H), and 5680001(I, J) treated with DMSO, 5 µM or 10µM of 827171 (A, C, E, G, I, respectively) and AZD7648 (B, D, F, H, J, respectively) at 0 h and the final time point of each experiment. Scale bar denotes 400 µm. The clustering reduces fluorescence intensity, but the cells are healthy, and the Incucyte fluorescence measurement does not decrease with treatment with 827171, in contrast to the treatment with AZD7648.
